# Human milk oligosaccharides in milk of mothers with term and preterm delivery at different lactation stage

**DOI:** 10.1016/j.carbpol.2023.121263

**Published:** 2023-12-01

**Authors:** Chuncui Huang, Yue Lu, Lin Kong, Zhendong Guo, Keli Zhao, Zheng Xiang, Xinyue Ma, Huanyu Gao, Yongfang Liu, Zhongmin Gao, Lijuan Xu, Wengang Chai, Yan Li, Yao Zhao

**Affiliations:** a Key Laboratory of Epigenetic Regulation and Intervention, Institute of Biophysics, Chinese Academy of Sciences, 15 Datun Road, Beijing 100101, China; b National Clinical Research Center for Child Health and Disorders, China International Science and Technology Cooperation base of Child development and Critical Disorders, Ministry of Education Key Laboratory of Child Development and Disorders, Chongqing Key Laboratory of Child Infection and Immunity, Children's Hospital of Chongqing Medical University, Chongqing 400015, China; c University of Chinese Academy of Sciences, 19 Yuquan Road, Beijing 100049, China; d Western Institute of Health Data Science, 28 High Tech Avenue, Chongqing 401329, China; e Glycosciences Laboratory, Faculty of Medicine, Imperial College London, London W12 0NN, United Kingdom

**Keywords:** Human milk oligosaccharides (HMOs), Duration of gestation, Distinctive oligosaccharides, Preterm delivery, Phenotype

## Abstract

Human milk oligosaccharides (HMOs) are structurally diverse unconjugated glycans, and play crucial roles in protecting infants from infections. Preterm birth is one of the leading causes of neonatal mortality, and preterm infants are particularly vulnerable and are in need of improved outcomes from breast-feeding due to the presence of bioactive HMOs. However, studies on specific difference in HMOs as a function of gestation time have been very limited. We established an approach to extract and analyze HMOs based on 96-well plate extraction and mass spectrometry, and determined maternal phenotypes through distinctive fragments in product-ion spectra. We enrolled 85 women delivering at different gestation times (25–41 weeks), and observed different HMOs correlating with gestation time based on 233 samples from the 85 donors. With the increase of postpartum age, we observed a regular changing trajectory of HMOs in composition and relative abundance, and found significant differences in HMOs secreted at different postpartum times. Preterm delivery induced more variations between participants with different phenotypes compared with term delivery, and more HMOs varied with postpartum age in the population of secretors. The sialylation level in mature milk decreased for women delivering preterm while such decrease was not observed for women delivering on term.

## Graphical abstract

**Figure:**
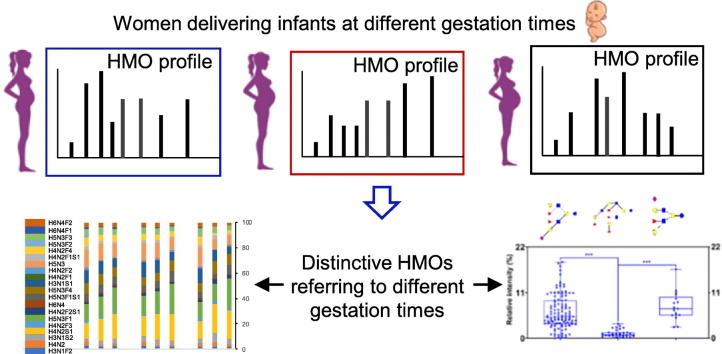

## 1 Introduction

Human milk is the natural food for infants after birth and contains a variety of components such as proteins, lipids, vitamins and carbohydrates, all of which provide nutrition as well as protection against infectious diseases, thus supporting the healthy growth and development of infants (Labbok et al., 2004; Leung & Sauve, 2005; Musilova et al., 2014). Human milk oligosaccharides (HMOs) are one of the most abundant components in human milk and modulate the activities of immune cells within the intestinal mucosa, and are therefore critical for establishing a healthy gut microbiome in infants (Boehm & Stahl, 2007; Dai et al., 2000; Newburg et al., 2005). >200 oligosaccharides have been identified in human milk. HMOs can also promote the growth of specific strains of beneficial bacteria such as bifidobacteria within the infant gastrointestinal tract, protecting the infant from colonization by pathogenic bacteria (De Leoz et al., 2015). HMOs, especially the fucosylated species, act as floating receptor analogs through competing for bacterial binding in the intestinal lumen to prevent intestinal pathogen adhesion to the epithelial surface (Newburg & Grave, 2014). Sialyated HMOs have been reported to play important roles in early brain growth (Wang, 2012).

Infants delivered before 37 weeks gestation are considered premature, with infants delivered after <28 weeks being classified as ‘very preterm infants’, between 28 and 32 weeks as ‘early preterm infants’, and between 32 and 36 weeks as ‘medium preterm infants’. Preterm infants are at an increased risk for necrotizing enterocolitis (NEC), sepsis, pneumonia, and neurodevelopment delay, primarily due to immaturity and dysfunction of essentially every component of their innate immune system. It has been also reported that HMOs modulate the risk for preterm birth (Pausan et al., 2020), and premature infants fed with mother milk exhibit lower rates of NEC and sepsis than those who are nursed with commercial formulated animal-derived milk powder (Underwood et al., 2015).

Detailed analysis of preterm milk components revealed some difference from those of term milk, including HMOs (Ballard & Morrow, 2013; Gidrewicz & Fenton, 2014). It has been reported in one previous study that HMOs containing α-1,2 linked Fuc were reduced in concentration in preterm milk during the first month of lactation, and the concentration of sialylated oligosaccharides was also different in preterm milk, in particular 3′-sialyllactose concentration was elevated (Austin et al., 2019). This study also showed that colostral preterm milk contained larger amounts of HMOs than term milk, with decreasing amounts of oligosaccharides during the course of lactation (Austin et al., 2019). However, studies assessing the composition and structural diversity of HMOs in milk from women delivering at different gestational periods have been very limited. Importantly, few distinct HMOs have been identified for different duration of gestation and different maternal phenotypes. This information is critical, however, for better understanding the mechanisms responsible for the bioactivity of HMOs and infections by pathogenic microorganisms.

HMOs are un-conjugated complex carbohydrate molecules synthesized in the mammary gland throughout lactation. Core structures are produced through the elongation of lactose with residues of galactose (Gal) and *N-*acetylglucosamine (GlcNAc), which could be further decorated with sialic acid (Neu5Ac) residues by the action of sialyltransferases and fucose (Fuc) residues by the function of fucosyltransferases. Unlike the polypeptide sequences oligosaccharides are highly branched with different linkages (Bode, 2006; De Leoz et al., 2012). Their backbones are frequently decorated with Fuc and NeuAc residues to form various peripheral recognition motifs, including the blood group-related ABO(H) and Lewis antigens. Fucosyltransferase 2 (FUT2) expressed in the mammary gland catalyzes the transfer of a Fuc residue to form an α1,2-linkage in the oligosaccharide present in human milk. FUT 2 is known as a “secretor” gene due to its role in expression of ABH(O) blood groups in various blood fluids (Bai et al., 2018). Fucosyltransferase 3 (FUT3) encoded by the Le gene determines the presence of α1,3/4-linked fucose residues on HMOs. Women with an active Le locus are classified as Lewis positive (Le^+^). FUT3 is involved in the formation of Lewis epitopes which play crucial roles in the bioactivity of HMOs (Pendu, 2004). One study proposed that infants who were breast-fed by women of a specific Lewis phenotype and/or secretor status were better protected against certain infections than other infants (Blank et al., 2011). In addition, it has been recently suggested that maternal phenotype may provide strong predictive biomarkers of adverse outcomes in premature infants (Morrow et al., 2011). Characteristic features of HMOs from mothers with different phenotypes are worth more detailed investigation, especially for women delivering preterm infants.

Better understanding the bioactivities of HMOs requires a series of techniques for oligosaccharide purification and analysis (including detection, characterization and quantification). Methods based on nuclear magnetic resonance (NMR), high performance liquid chromatography (HPLC) coupled with mass spectrometry (MS) and capillary electrophoresis (CE) have been developed to analyze HMOs (Hageman et al., 2018; Lang et al., 2021; Mank et al., 2020; van Leeuwen et al., 2014). Prior to using these techniques, purification of oligosaccharides is extremely challenging as the physical properties of oligosaccharides closely resemble those of highly abundant lactose. Residual lactose may preclude instrumental analysis of HMOs, and therefore removal of large amount of lactose is necessary (Robinson et al., 2018). In addition, it is essential to eliminate lactose when investigating biological functions of oligosaccharides, as it often interferes with specific functions of HMOs such as prebiotic activities. One commonly used method to separate milk oligosaccharides from lactose involves the use of graphitized carbon (GC) with appropriate modification of established solid phase extraction (SPE) conditions (Robinson et al., 2018). An additional advantage of GC is the excellent retention characteristics in the context of oligosaccharide desalting and preconcentration (Davies et al., 1992; Koizumi et al., 1991; Marx et al., 2014; Redmond & Packer, 1999).

Here, we set out to investigate HMOs from women delivering at different gestation times using an established approach including HMO extraction and analysis. Maternal phenotypes were determined through distinctive fragments generated in the product-ion spectrum of diagnostic milk oligosaccharides for phenotype determination using MALDI-TOF MS/MS. In summary, significantly different HMOs were observed among women delivering at different gestation times, and more variations in HMOs as a function of gestation time were found in secretor participants.

## 2 Materials and methods

### 2.1 Materials

Bovine serum albumin (BSA), standard peptides for mass calibration, sodium chloride, acetic acid, sodium hydroxide and trifluoroacetic acid were purchased from Sigma-Aldrich (St. Louis, MO). Acetonitrile, ethanol, methanol and water were obtained from Avantor Performance Materials (Center Valley, PA). 2,5-Dihydroxybenzoic acid was purchased from ProteoChem (Hurricane, UT). C18-Sep-Pak 96-well plates were obtained from Waters (Milford, MA). Graphitized carbon solid phase extraction cartridges were obtained from Acchrom Tech (Beijing, China), and graphitized carbon 96-well plates were designed and provided by Sipore (Dalian, China) as required. All the chemicals were of analytical grade or better, and used without further purification.

### 2.2 Trial design and human milk collection

In this study, human milk was longitudinally collected from women delivering infants at different gestation times (25–41 weeks) (Table 1). Neonatal demographic and delivery data were collected from the medical charts upon enrollment in Children's Hospital of Chongqing Medical University. Human milk was collected from women in the southwest area of China with the delivery date from 2019 to 2021. Colostrum was collected immediately after delivery, transitional milk was collected on day 7 post delivery, and mature milk was collected after day 15 post delivery (20–30 days). Milk samples were collected and homogenized, and 2 mL was transferred to a graduated freezing tube. All samples were stored at −80 °C until analysis.

**Table 1:** Details for human milk samples.

Samples	Participants	Lactation			
		Colostrum	Transitional milk	Mature milk	Total
Gestation times (Total)	85 (100 %)	82	64	87	233 (100 %)
<28 week	7 (8.2 %)	8	10	12	30 (12.9 %)
28–32 week	19 (22.4 %)	17	20	24	61 (26.2 %)
33–36 week	28 (32.9 %)	33	22	26	81 (34.8 %)
≥37 week	31 (36.5 %)	24	12	25	61 (26.2 %)
Phenotypes (Total)	85(100 %)	82	64	87	233 (100 %)
Secretor (Se^+^)	69(81.2 %)	67	51	70	188 (80.7 %)
Non-secretor (Se^−^)	16(18.8 %)	15	13	17	45 (19.3 %)
Lewis positive (Le+)	77 (90.6 %)	44	37	48	129 (55.4 %)
Lewis negative (Le-)	8 (9.4 %)	38	27	39	104 (44.6 %)
Se^+^Le^+^	61 (71.8 %)	28	24	31	83 (35.6 %)
Se^+^Le^−^	8 (9.4 %)	38	27	39	104 (44.6 %)
Se^−^Le^+^	16 (18.8 %)	16	13	17	46 (19.7 %)

### 2.3 Extraction of oligosaccharides

Oligosaccharides were extracted from human milk samples as previously described (Robinson et al., 2018). Briefly, 100 μL aliquots of the milk samples collected from women were transferred into tubes, and diluted with equivalent water. Samples were then centrifuged at 4500*g* and 4 °C for 20 min, and the middle layer containing oligosaccharides was transferred to a new tube. Milk lipids in the upper layer and precipitates at the bottom were discarded. Two volumes of ethanol were added to the transferred skim milk, and the mixture was incubated at 4 °C for 2 h with constant stirring. Subsequently, the sample was centrifuged at 4500*g* and 4 °C for 20 min, and the supernatant was transferred to a new tube followed by vacuum evaporating (LABCONCO CentriVAP, MO).

### 2.4 Purification of oligosaccharides and removal of lactose

Dried oligosaccharides were dissolved in water, and divided into two aliquots followed by vacuum evaporating. One aliquot dissolved in 5 % acetic acid was first purified by C18 96-well plate to remove residual proteins, and was further purified by GC SPE column or GC 96-well plate to remove lactose. The oligosaccharides dissolved in 5 % acetic acid were applied to C18 96-well plate which was pre-conditioned successively with methanol, 5 % acetic acid, propan-1-ol and 5 % acetic acid (3×). Oligosaccharides containing lactose were collected in 5 % acetic acid, and lyophilized in a clean screw-capped glass culture tube. Dried oligosaccharides were dissolved in 0.1 % TFA, and then applied to the GC SPE column or GC 96-well plate which was preconditioned by 80 % ACN in 0.1 % TFA. Lactose-free oligosaccharides were then eluted stepwise by 10 % ACN/ 0.1 % TFA and 20 % ACN/0.1 % TFA. To remove any remaining proteins, lipids, peptides, and lactose, for comparison, the second aliquot sample was purified directly by GC SPE column or GC 96-well plate.

### 2.5 Permethylation of HMOs

Permethylation and subsequent purification of permethylated HMOs were performed as previously reported (Dell et al., 1994; Sun et al., 2018). Briefly, methyl iodide was added to HMOs in the presence of NaOH slurry, and the sample was then agitated on an automatic shaker at room temperature for 20 min. Chloroform was added and mixed thoroughly with the sample. The mixture was then allowed to settle into two layers. The upper aqueous layer was removed, and the chloroform layer was dried under a gentle stream of nitrogen. Finally, the mixture was purified on the C18 Sep-Pak cartridge.

### 2.6 MALDI-TOF MS and identification of HMO structures

Permethylated HMOs were analyzed on an Axima MALDI Resonance mass spectrometer with a QIT-TOF configuration (Shimadzu, Japan). Each sample was spotted triplicate. A nitrogen laser was used to irradiate samples at 337 nm, with an average of 500 shots accumulated at all the crystallization sites. For reproducible analysis, each sample was scanned for ten times, and average relative intensities were obtained. Permethylated HMOs dissolved in methanol were applied to a μfocus MALDI plate target (900 μm, 384 circles, Hudson Surface Technology, NJ). A matrix solution (0.5 μL) of 2,5-dihydroxybenzoic acid (20 mg/mL) in a mixture of methanol/water (1:1) containing 0.1 % trifluoroacetic acid and 1 mM NaCl was added to the plate and mixed with samples. The samples were air dried at room temperature before analysis, and primary MS and MS^2^ production-ion spectra were initially acquired.

HMO structures were identified using our previously developed GIPS and GIPS-mix strategy (Sun et al., 2018; Huang et al., 2023). Briefly, fragment ions in the MS^2^ spectrum with relative intensity above 10 % were selected as precursors for MS^3^ scanning, and all the MS^2^ and MS^3^ spectra were fed into GIPS platform as mzXML files. If HMO structures were identified, the identification process terminated. Otherwise, further analysis and MS^n^ scanning were carried out until HMO structures were identified.

### 2.7 Statistical analysis

Multivariate statistical analysis and univariate statistical analysis were both performed in the present study. The MALDI MS data were extracted and normalized using MarkerView 1.2.1.1. The monoisotopic peaks of identified oligosaccharide were selected and the intensities were normalized using the total intensity. The normalized data were used for further statistical analysis. Data were presented as means ± SD, and *p* values lower than 0.05 were considered statistically significant.

In multivariate statistical assay, we performed orthogonal partial least squares-discriminant analysis (OPLS-DA) which has been largely used in the metabolomics context, and is now the multivariate linear model of choice for classification/discrimination (Senizza et al., 2023). In the present study, OPLS-DA was used for discriminant analysis of HMOs at different gestation times, stages of lactation, and phenotypes. Specific oligosaccharides were filtrated between groups with significant difference by the value of variable importance for projection (VIP) and *p* value. In univariate statistical analysis, difference analysis was performed using IBM SPSS Statistics 23 software after importing relative intensities of HMOs. We first performed normal distribution test and homogeneity test of variance of each oligosaccharide, and then performed *t*-test or Bonferroni multiple comparison on HMOs satisfying these two tests. Otherwise, nonparametric test was accordingly conducted. Kruskal-Wallis ANOVA test (Theodorsson-Norheim, 1986) and Mann-Whitney test (Perme & Manevski, 2019) were then performed for multi-group and two-group test respectively.

## 3 Results and discussion

### 3.1 Extraction of HMOs

Graphitized carbon solid phase extraction is a well-established technique commonly used for glycan extraction and purification. In order to improve the extraction efficiency and throughput, four kinds of columns including GC columns, GC 96-well plates, C18 96-well plates in combination with GC columns, and C18 96-well plates in combination with GC 96-well plates were evaluated. As shown in Fig. S1 and Table S1, approximately equivalent amounts of proteins were removed from the milk samples following extraction using four SPE columns. These results demonstrated that GC was highly efficient in removing proteins regardless of the combination of C18 columns. Considering the efficiency and high throughput, C18 columns were discarded, and GC columns as well as GC 96-well plates were further evaluated.

**Fig. 1:**
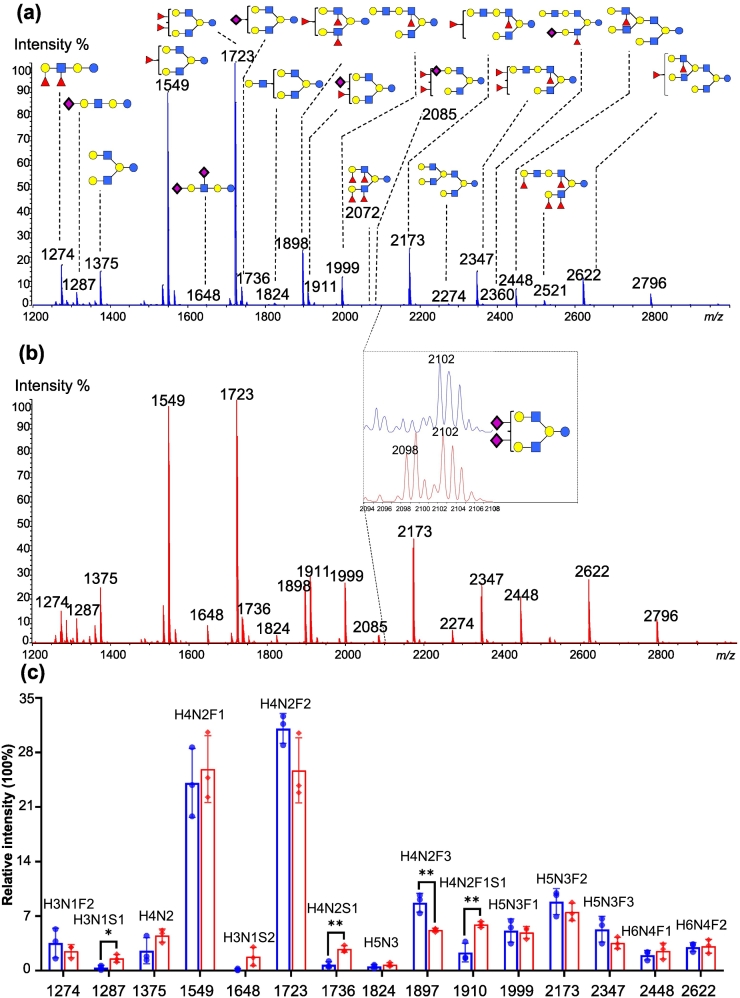
MALDI-TOF profiles of HMOs extracted using GC columns (a) and GC 96-well plates (b), shown with the corresponding relative quantification results (c) (GC columns in blue, GC 96-well plates in red). Error bars represent the standard deviation (SD) of all the analysis (*n* = 10). *, 0.01 ≤ *p* < 0.05, **, 0.001 ≤ *p* < 0.01. (For interpretation of the references to colour in this figure legend, the reader is referred to the web version of this article.)

As shown in Fig. S2, peaks corresponding to lactose were not observed in the mass spectrum of oligosaccharide sample flowing through GC 96-well plate, while intensive peaks of lactose were detected in the spectrum of oligosaccharide sample that was not applied on GC 96-well plate. The result indicated that lactose was extensively retained on GC 96-well plates. While 42 HMOs were detected in the MS spectrum of the sample extracted using GC 96-well plates, only 39 HMOs were detected in the sample extracted using GC columns (Table S2). Specifically, three sialylated HMOs were additionally extracted using GC 96-well plates. We identified oligosaccharide branching patterns based on multistage MS scanning and the intelligent GIPS platform previously developed in our laboratory (Sun et al., 2018; Huang et al., 2020; Huang et al., 2023), and the identified structures were shown in Fig. 1. In addition, based on relative intensity of HMOs extracted using GC columns and GC 96-well plates, significant differences were not detected for most HMOs except H4N2F3, H3N1S1, H4N2S1 and H4N2F1S1(Fig. 1c and Table S3). The clear increase in relative intensity of the three sialylated HMOs extracted by GC 96-well plates was due to the diminished loss of labile bonds linking sialic acid during sample processing, which agreed with previous report (Totten et al., 2014).

Together, these results indicated that HMOs extracted using GC columns and GC 96-well plates were approximately identical, and more acidic HMOs were extracted using GC 96-well plates. On basis of these results, we utilized 96-well plates packed with GC for the following analysis of large amounts of human milk samples.

### 3.2 Determination of maternal phenotypes

Unique oligosaccharides present in human milk can determine maternal phenotypes as they carry distinctive residues that are transferred under the catalyzation of specific enzymes and genes. In order to investigate the secretor status of women, we detected the presence of LNFP-I which carried the α1,2-linked fucose residue in non-reducing terminus. Furthermore, we determined the Lewis blood group type corresponding to Le gene by the presence of LNFP-II/LNFP-III which has an α1,4/3-linked fucose residue attached to the sub-terminal GlcNAc. LNFP-I, LNFP-II and LNFP-III were indicated by the molecular mass [M + Na]^+^ at *m*/*z* 1100 (M: Fuc1.Hex3.HexNAc1) in the primary mass spectrum. However, multiple isomeric oligosaccharides with m/z 1100 exist in human milk, and the frequent resolution is separating these isomers by chromatography, which is challenging and ambitious. In order to identify LNFP-I, LNFP-II and LNFP-III in large numbers of human milk samples in high throughput, we proposed to investigate distinctive fragments produced from dissociation of precursor ions at *m*/*z* 1100.

**Fig. 2:**
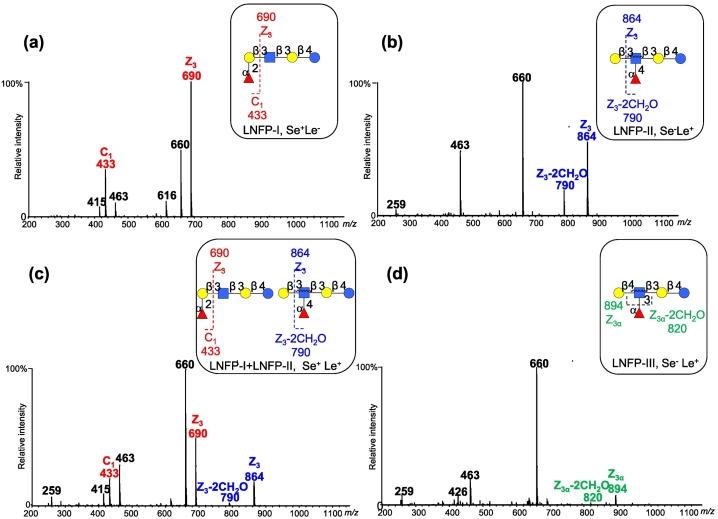
Product-ion mass spectra of HMOs with m/z 1100 generated using MALDI-TOF MS/MS. Distinctive fragment ions were observed at m/z 433 and 690 (a), at m/z 790 and 864 (b), at m/z 433, 690, 790 and 864 (c), and at m/z 820 and 894 (d).

We found that unique fragment ions at m/z 433 and 690 were characteristic for LNFP-I, and distinctive fragment ions at *m*/*z* 790 and 864 were diagnostic for assignment of LNFP-II, and distinctive fragment ions at *m*/*z* 820 and 894 were diagnostic for assignment of LNFP-III. These results were in agreement with those from our previous study on epitope identification (Huang et al., 2020). As shown in Fig. 2, MALDI-MS/MS fragmentation of molecules at m/z 1100 in human milk resulted in four types of mass spectra. The characteristic fragments of α1,2-linked fucose residue at m/z 433 and 690 in Fig. 2a and c were indicative of the phenotype of secretors (Se^+^). In addition, we found unique fragment ions at m/z 790 and 864 in Fig. 2b and c, which indicated the attachment of α1,4-linked fucose residue to the pentasaccharide. Unique fragment ions at m/z 820 and 894 in Fig. 2d indicated the attachment of α1,3-linked fucose residue. The α1,4/3-linked fucose residue was transferred with the action of FUT3 encoded by the Le gene, and therefore the presence of this residue indicated the maternal phenotype as Lewis positive (Le^+^). The absence of distinctive fragments for α1,2-linked and α1,4/3-linked fucose residues resulted in the identification of nonsecretor (Se^−^) and Lewis negative (Le^−^) phenotype.

Next, we determined phenotypes of all enrolled delivery women based on these distinctive fragments. As listed in Table 1, we detected 69 secretors among the enrolled women, and we found 77 women were Le positive. Combined the Se and Le gene, we assigned 61 out of 85 enrolled women to the Se^+^Le^+^ group, which constituted 71.8 % of the total women assessed (Table 1). Of all enrolled women, 18.8 % were found to be of Se^−^Le^+^ phenotype, while 9.4 % of the women were assigned to be with Se^+^Le^−^ phenotype. However, we found that none of the enrolled women exhibited Se^−^Le^−^ phenotype. These findings on distributions of phenotypes agree with previous reports estimated in Chinese mothers although phenotypes vary among populations in different regions and ethnic groups (Elwakiel et al., 2018; Li et al., 2022).

### 3.3 Difference of HMOs between women with preterm delivery and term delivery

**Fig. 3:**
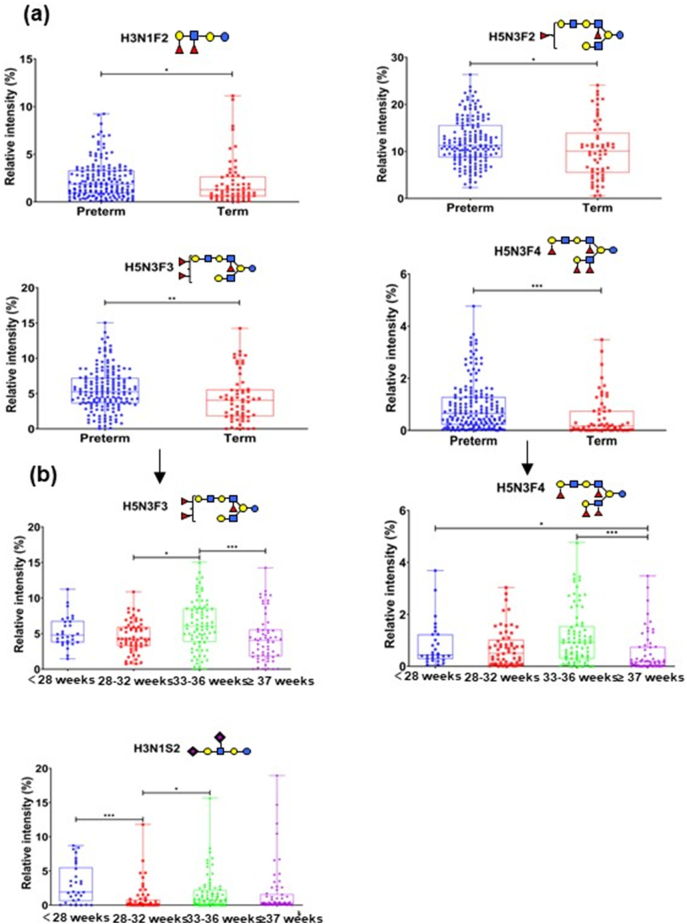
Relative abundance of HMOs from women with preterm delivery (<37 weeks) and women with term delivery (≥37 weeks) (a). Relative abundance of HMOs at different gestation times (b). *, 0.01 ≤ *p* < 0.05；**, 0.001 ≤ *p* < 0.01；***, *p* < 0.001.

To investigate the difference on HMOs between women with preterm delivery (<37 weeks) and term delivery (≥37 weeks), we statistically calculated the relative intensity of each oligosaccharide in the MALDI mass spectrum as previously reported (Sun et al., 2019). The reproducibility of relative intensity of HMOs was first assessed by repeated scanning, and the relative standard deviation (RSD) of the representative HMOs was satisfying (Fig. S3, Table S4), indicating that statistically calculating the relative intensity of HMOs in the MALDI mass spectrum was reliable. Four HMOs including H3N1F2, H5N3F2, H5N3F3 and H5N3F4 differed significantly when comparing women delivering preterm versus women delivering on term (Fig. 3a). We found that these four HMOs were all fucosylated neutral oligosaccharides with multiple fucose residues attached to their backbones, and the relative abundance of these fucosylated HMOs was higher for women delivering preterm infants.

In order to extensively investigate the difference in HMOs between women with preterm delivery and term delivery, enrolled women were divided into subgroups according to their phenotypes. Among the significantly different four HMOs between women with preterm delivery and women with term delivery, we further found that three HMOs including H3N1F2, H5N3F3 and H5N3F4 differed significantly in the population of secretors (Fig. S4). No significant difference was detected between nonsecretors delivering preterm and nonsecretors delivering on term (data not shown). These observations suggested that more variations occurred between preterm delivery and term delivery for secretors. Since the observed different HMOs in secretors are multi-fucosylated, fucosylated HMOs are likely to be more susceptible to the duration of gestation for secretors inherently carrying FUT 2 gene compared with nonsecretors.

**Fig. 4:**
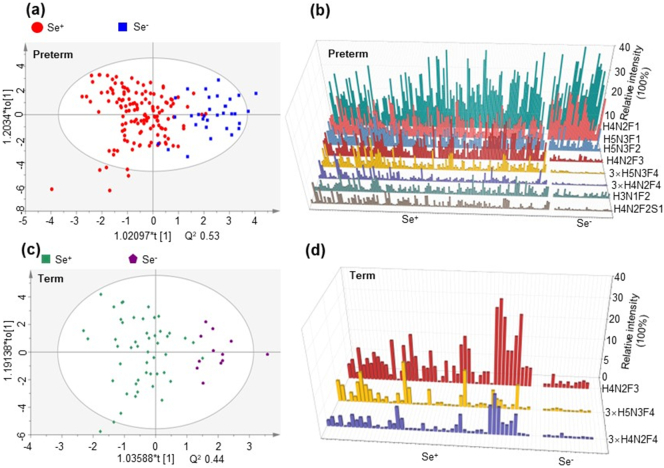
OPLS-DA predictive vs orthogonal scores for secretors and nonsecretors delivering preterm infants (a), and the identified significantly different HMOs (b). OPLS-DA predictive vs orthogonal scores for secretors and nonsecretors delivering term infants (c), and the identified significantly different HMOs (d). The OPLS-DA analysis was based on the 20 most abundant oligosaccharides in the milk.

We also detected more significantly different HMOs between secretors and nonsecretors in the group of preterm delivery than the group of term delivery. As shown in Fig. 4a and Table S5, 8 HMOs including H3N1F2, H4N2F1, H4N2F3, H5N3F1, H4N2F4, H4N2F2S1, H5N3F2 and H5N3F4 differed significantly between secretors and nonsecretors for women delivering preterm. However, when detecting significantly different HMOs in the group of term delivery, we found that only three HMOs differed significantly in relative intensity between secretors and nonsecretors. These findings suggested that more HMOs differed between secretors and nonsecretors for women delivering preterm infants.

Furthermore, we divided the enrolled women by secretor gene and Le gene to form three subgroups including Se^+^Le^+^, Se^−^Le^+^ and Se^+^Le^−^ (Table 1). As shown in Fig. S5, H4N2F3, H4N2F4, H4N2F2S1 and H5N3F4 were found to be significantly different in four phenotypes for women delivering on term. These four HMOs all differed significantly between phenotype Se^+^Le^+^ and Se^−^Le^+^, while only H4N2F4 differed significantly between phenotype Se^+^Le^+^ and Se^+^Le^−^. Next, we compared HMOs in different phenotypes for women delivering preterm. Eleven HMOs were significantly different in three phenotypes for women with preterm delivery, suggesting that more HMOs differed among phenotypes for women delivering preterm than women delivering on term. Again, these finding indicated that preterm delivery induced more alternations in HMOs for women with different phenotypes, and the genes relating to phenotypes were susceptible to the duration of gestation.

In summary, significantly different HMOs were observed between women with preterm delivery and term delivery, and more HMOs differed among phenotypes for women with preterm delivery. These results indicated that preterm delivery resulted in more disparity in HMOs for women with different phenotypes.

### 3.4 Difference of HMOs between women delivering at different gestation times

In order to explore in detail whether different duration of gestation affect the synthesis of oligosaccharides, we further statistically studied the relative abundance of HMOs in groups classified based on duration of gestation. As shown in Fig. 3b, H5N3F3 and H5N3F4, which have been found to be significantly different between women with preterm delivery and term delivery (Fig. 3a), further showed significant difference among women delivering at different gestation times. Specifically, H5N3F3 was significantly different between women delivering infants at weeks 28–32 and women delivering infants at weeks 33–36, and significant difference in H5N3F3 was also found between women delivering infants at weeks 33–36 and women with term delivery (≥37 weeks). Detailed analysis considering gestation time manifested that H5N3F4 differed significantly in relative intensity between women delivering infants at weeks 33–36 and women with term delivery. Significant difference in H5N3F4 was also observed in women with very early preterm delivery (<28 weeks) and women with term delivery (≥37 weeks). In addition, we found DSLNT denoted as H3N1S2 was significantly different between women in very early preterm delivery (<28 weeks) and women delivering infants at weeks 28–32, and also between women delivering infants at weeks 28–32 and women delivering infants at weeks 33–36. (Fig. 3b).

Together, these results suggested that HMOs were affected by the duration of gestation, and preterm infants soaked up unequal HMOs compared with term infants. As a result, the gut microbiome in breast-fed preterm infants might be different from that of term infants, and the ability to resist virus and bacterium infection was not identical for exclusively breast-fed preterm and term infants.

### 3.5 Dynamic changes of HMOs during lactation for women delivering at different gestation times

In order to explore the distinguishing features in HMO changes for women who gave birth after different gestation times, we measured dynamic changes of HMO compositions during lactation.

**Fig. 5:**
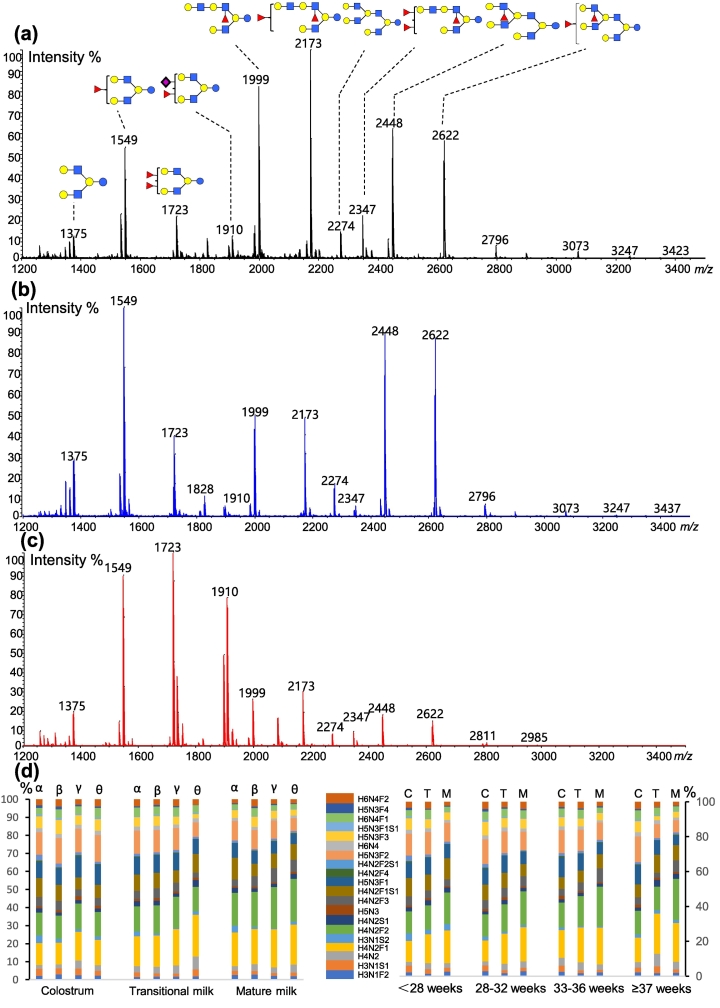
MALDI-TOF profiles of HMOs present in colostrum (a), transitional milk (b) and mature milk (c). HMO compositions at the same postpartum ages (the period of colostrum, transitional milk and mature milk respectively) for the enrolled participants delivering at different gestation times (d, left panel), and HMO levels during breast feeding as a function of gestation time (d, right panel). In the left panel of d, α refers to women delivering before 28 weeks, β refers to women delivering between 28 and 32 weeks, and γ refers to women delivering between 33 and 36 weeks, and θ refers to women delivering after 37 weeks. In the right panel of d, C refers to colostrum, T refers to transitional milk, M refers to mature milk. HMOs in annotation were arranged by the increase of molecular mass (bottom-up, from H3N1F2 to H6N4F2). 100 % in (a), (b), and (c) refers to the relative intensity of the most intensive peaks in the mass spectra, and 100 % in (d) refers to the sum of relative intensity after normalization for 20 most abundant HMOs in each group.

As shown in Fig. 5, HMOs at different lactation stages exhibited distinguishing profiles in mass spectrum, and HMO structures were assigned from compositional information inferred from their *m*/*z* values considering biosynthetic pathway and multistage MS dissociation. Based on the analysis of representing HMOs in high abundance, we first observed that HMOs with large molecular mass were more abundant in the colostrum. Furthermore, the relative abundance of large HMOs gradually decreased as a function of lactation time. In comparison, low molecular mass HMOs gradually increased with lactation time, and constituted the main components in mature milk. In consideration of gestation time, similar gradually changing trajectory of HMOs was observed for women who gave birth at different gestation times (Fig. 5d). Together, these results indicated that the dynamic changing trajectory considering molecular mass during lactation was identical for all the women regardless of the difference in duration of gestation although significantly different HMOs were observed between women with preterm delivery and term delivery.

**Fig. 6:**
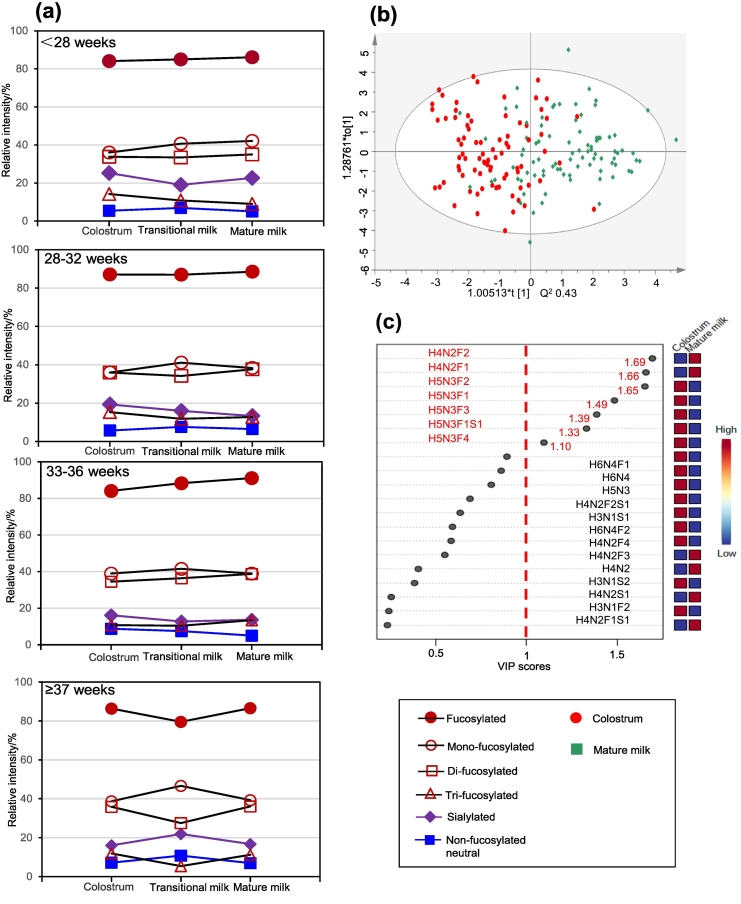
Dynamic changes of HMOs with certain types during lactation for women delivering at different gestation times (a), and discriminant analysis on different lactation stages through HMOs (b), and analysis on the value of variable importance for the projection for specific HMOs (c). The OPLS-DA analysis was based on the 20 most abundant oligosaccharides in the milk.

In order to investigate dynamic changes of HMOs with certain types during lactation, we further divided oligosaccharides as fucosylated HMOs, mono−/di−/tri-fucosylated HMOs, sialylated HMOs and non-fucosylated neutral HMOs. When we assessed fucosylated HMOs in the milk, we found that the relative abundance of fucosylated HMOs was higher in mature milk than that in colostrum for all women, despite a slight decrease of fucosylated HMOs observed in transitional milk of women with term delivery (≥37 weeks). In comparison, we found that sialylation levels of HMOs in colostrum and mature milk were nearly identical following an increase in transitional milk for women delivering after 37 weeks (Fig. 6a). However, for women delivering before 37 weeks, sialylation levels in mature milk and transitional milk were lower than that in colostrum. A previous study using an ex vivo model with whole human blood reported that sialylation HMOs reduced platelet-neutrophil complex (PNC) formation and subsequent neutrophil activation (Bode et al., 2004). Another study found that in human milk, sialylated HMOs produced the majority of Sia. Also, Sia is one of the sources of Sia-containing gangliosides and poly-Sia containing glycopeptides, all of which are essential for brain development and cognition (Wang, 2012). When we assessed the non-fucosylated neutral HMOs from women delivering at different gestation times, we did not find significant differences.

To further test specific different HMO during lactation for all the women delivering at different gestation times, we first used an OPLS-DA assay. As shown in Fig. S6, colostrum and transitional milk were not clearly differentiated, and differentiation of transitional milk from mature milk could not be achieved either. However, we observed a transitional tendency in HMOs from colostrum to mature milk. Furthermore, we found unambiguous differentiation between colostrum and mature milk in further OPLS-DA analysis (Fig. 6b), indicating that HMO components were different in colostrum and mature milk. As shown in Fig. 6c, seven HMOs, namely H4N2F2, H4N2F1, H5N3F2, H5N3F1, H5N3F3, H5N3F1S1 and H5N3F4 were characterized by high VIP values, indicating these HMOs were different in colostrum and mature milk for women delivering at different gestation times.

Together, these results clearly demonstrated that distinctive HMOs were present in specific lactation stage, and further analysis based on specific gestation time could be performed.

### 3.6 Distinct HMOs during lactation for women delivering at different gestation times

**Fig. 7:**
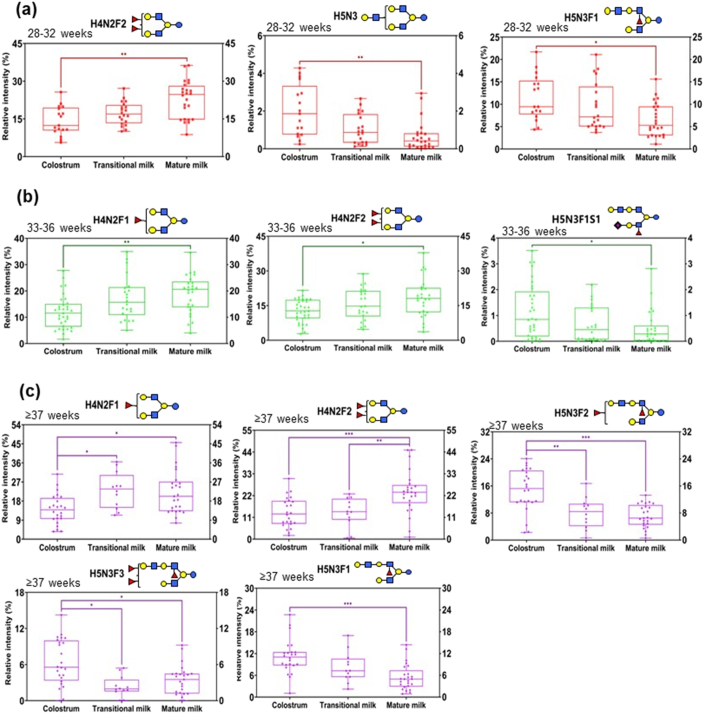
Distinctive HMOs for women delivering at different gestation times. Women delivering at 28–32 weeks (a), 33–36 weeks (b), and after 37 weeks (c). *, 0.01 ≤ *p* < 0.05；**, 0.001 ≤ *p* < 0.01；***, *p* < 0.001.

In order to find unique HMOs during lactation for women delivering at specific gestation time, we further investigated HMOs in the subgroups of women with different duration of gestation. As shown in Fig. 7, for women delivering infants at weeks 28–32, three HMOs including H4N2F2, H5N3 and H5N3F1 differed significantly between colostrum and mature milk. In addition, H4N2F2 dynamically increased from colostrum to mature milk, while H5N3 and H5N3F1 gradually decreased from colostrum to mature milk. Three HMOs including H4N2F1, H4N2F2 and H5N3F1S1 were determined to be significantly different between colostrum and mature milk for women delivering infants at weeks 33–36. When we analyzed HMOs from women delivering before 37 weeks, we found that five differed significantly between colostrum and mature milk. Critically, these oligosaccharides were all fucosylated. These findings indicated that HMOs differed in the milk secreted at different postpartum times, and a regular and dynamic program defined the HMO trajectory following the prolong of postpartum time.

One previous work reported that DSLNT protected infants from developing NEC, and the concentration of DSLNT in maternal milk may be a non-invasive biomarker to identify breast-fed infants at risk of developing NEC (Jantscher-Krenn et al., 2012). We have found that DSLNT differed between women delivering at different gestation times (denoted as H3N1S2 in Fig. 3b). However, the relative abundance of DSLNT in each lactation stage remains to be unknown, which is crucial for infant-feeding, especially for preterm infants. In order to obtain the relative abundance of DSLNT during lactation, we investigated dynamic changes during lactation for women with different duration of gestation. As shown in Fig. S7, the relative abundance of DSLNT in mature milk was lower than that in colostrum despite the uncertain changing tendency in transitional milk. Interestingly, among all the women delivering at different gestation times, the relative abundance of DSLNT in mature milk was highest for women delivering before 28 weeks. In consideration of the limited number of participants delivering before 28 weeks in the present study, further investigation needs to be explored with enlarged sample size. However, women with low-level DSLNT in mature milk need to pay more attention to breast-feeding.

### 3.7 Analysis of HMOs during lactation for women with different secretor status

As investigated above, different HMOs existed in lactation stages for women with different duration of gestation. In order to explore whether different HMOs during lactation are related to different phenotypes, we further investigated different HMOs during lactation in the group of secretors and nonsecretors.

We first comparatively studied levels of HMO with certain types in secretors and nonsecretors. As shown in Fig. S8, relative intensity of fucosylated HMOs in nonsecretors was higher than that in secretors, while sialylated HMOs were more abundant in secretors. The increased sialylated HMOs in secretors can help promoting immune responses and brain development for breast-fed infants (Wang, 2012). These results indicate that HMOs from secretors and nonsecretors were different, and detailed descriptions on differences of HMOs between secretors and nonsecretors were presented in Supplementary Text and shown in Figs. S9 and S10.

**Fig. 8:**
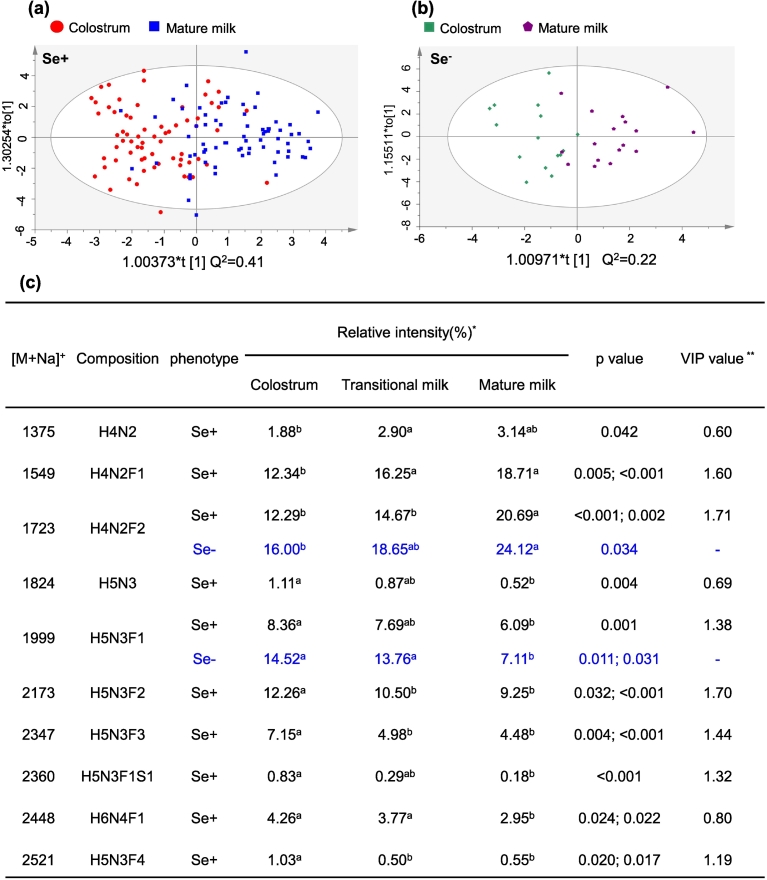
OPLS-DA assay for differentiation of colostrum and mature milk from secretors (a) and nonsecretors (b), and the detected significantly different HMOs in different lactation stages for secretors and nonsecretors (c). Multiple comparisons were performed between colostrum, transitional milk and mature milk. No significant difference was detected for the relative intensity figure labeled with the same superscript, and *p* value was not provided. Significant difference was detected for the relative intensity figure labeled with different superscript, and *p* value was presented. In panel (c), superscripts containing the same letters represent that significant difference was not found between these two groups, while superscripts containing different letters represent significant difference was observed in these two groups. The OPLS-DA assay was based on the 20 most abundant oligosaccharides in the milk.

Next, we analyzed different oligosaccharides in three lactation stages from secretors and nonsecretors individually using an OPLS-DA assay. As shown in Fig. 8a, colostrum and mature milk from secretors were differentiated, indicating that HMOs in colostrum and mature milk were different in the population of secretors. In comparison, we found that HMOs in colostrum and mature milk from nonsecretors only differed slightly (Fig. 8b). As shown in Fig. 8c, the relative abundance of ten HMOs differed significantly among different lactation stages for secretors. However, we only found two HMOs, H4N2F2 and H5N3F1, statistically differed during lactation for nonsecretors (Fig. 8c).

These findings demonstrated that more HMOs varied in relative abundance during lactation for secretors, and less variation in HMOs abundance was observed during lactation for nonsecretors. It is probably that enzymes and genes involved in the pathway of HMO synthesis actively worked as a function of lactation for secretors.

## 4 Conclusion

Significantly different HMOs were first observed between women with preterm and term delivery, and distinctive HMOs were further found to be present among women delivering at 25–41 weeks. The HMO trajectory in composition and relative abundance during lactation for women delivering at different gestation times was largely identical, suggesting that delivery induced a regular and dynamic program that defined the HMO trajectory during lactation. However, significantly different HMOs existed in milk secreted at different postpartum ages.

The different HMOs between women with preterm and term delivery were found largely in secretor rather than nonsecretor donors, and more HMOs varied during lactation for secretors than nonsecretors. These findings demonstrated preterm delivery triggered more variations for secretors, and HMOs derived from secretors were more seriously affected by postpartum ages. Enzymes and related genes involved in the pathway of HMOs synthesis seemed in more active status for secretors, and HMOs derived from secretors were more susceptible to maternal variations. Unfortunately, these observations have not been fully discussed in previous studies. Although the exact cause and effect of varied levels of prolactin with HMO production have not been sufficiently proven, its association with the different amounts or even structures of HMOs secreted at different gestation times may still need to be considered (Powe et al., 2011).

The sialylation level in mature milk decreased for women delivering preterm while such decrease was not observed for women delivering on term. For instance, the relative abundance of DSLNT in mature milk was detected to be lower than that in colostrum. In addition, the sialylated HMOs were more abundant in secretors than nonsecretors. The present work may provide a potential groundwork for further mechanistic studies on the inherent liability of preterm infants to virus infections, and also a foundation for development of “personalized” HMO additives for nutrition. The possible physiological significance of the observations remains to be determined. Systematic analysis aiming to clinical diagnosis and industrial production needs to be fully explored without limitation in sample collection and sample size.

## CRediT authorship contribution statement

C. Huang, Y. Li and Y. Zhao conceived the study. C. Huang and Y. Li designed the extraction methodologies, and established the analysis procedure. Z. Guo, K. Zhao, X. Ma and H. Gao performed the extraction and mass spectrometry experiments, and analyzed the mass spectral data. Y. Lu, L. Kong and Z. Xiang performed the enrollment of women, and collected human milk for analysis. Y. Liu, Z. Gao and L. Xu provided critical comments and advice on the biological functions of human milk on infant growth and health. W. Chai provided critical review on the methodologies and results. C. Huang, W. Chai, Y. Li and Y. Zhao wrote the manuscript. All authors discussed the results and commented on the manuscript.

## Human rights and informed consent

Human milk was collected on the approval of the Institutional Review Board, Children's Hospital of Chongqing Medical University (File No. 2019–220). All procedures performed in studies involving human participants were in accordance with the ethical standards of Biosafety and Ethics Committee in Institute of Biophysics, Chinese Academy of Sciences, and with the 1964 Helsinki Declaration and its later amendments or comparable ethical standards. Informed consent was obtained for experimentation from all the human subjects. The privacy rights of human subjects must always be observed.

## Declaration of competing interest

The authors declare no competing financial interest.

## Data Availability

Data will be made available on request.

## Appendix A Supplementary data

Supplementary material Image 1

## References

[bb0005] Austin S., De Castro C.A., Sprenger N., Binia A., Affolter M., Garcia-Rodenas C.L., Fumeaux C.J.F. (2019). Human milk oligosaccharides in the milk of mothers delivering term versus preterm infants. Nutrients.

[bb0010] Bai Y., Zhou J., Fan Q., Liu M., Hu Y., Xu Y., Zhang L., Yuan J., Li W., Ze X., Malard P., Guo Z., Yan J., Li M. (2018). Fucosylate human milk oligosaccharides and N-glycans in the milk of Chinese mothers regulate the gut microbiome of their breast-fed infants during different lactation stages. mSystems.

[bb0015] Ballard O., Morrow A.L. (2013). Human milk composition: Nutrients and bioactive factors. Pediatric Clinics of North America.

[bb0020] Blank D., Gebhardt S., Maass K., Lochnit G., Dotz V., Blank J., Geyer R., Kunz C. (2011). High-throughput mass finger printing and Lewis blood group assignment of human milk oligosaccharides. Analytical and Bioanalytical Chemistry.

[bb0025] Bode L. (2006). Recent advances on structure, metabolism, and function of human milk oligosaccharides. The Journal of Nutrition.

[bb0030] Bode L., Rudloff S., Kunz C., Strobel S., Klein N. (2004). Human milk oligosaccharides reduce platelet-neutrophil complex formation leading to a decrease in neutrophil beta 2 integrin expression. Journal of Leukocyte Biology.

[bb0035] Boehm G., Stahl B. (2007). Oligosaccharides from milk. The Journal of Nutrition.

[bb0040] Dai D.W., Nanthkumar N.N., Newburg D.S., Walker W.A. (2000). Role of oligosaccharides and glycoconjugates in intestinal host defense. Journal of Pediatric Gastroenterology and Nutrition.

[bb0045] Davies M., Smith K.D., Harbin A.M., Hounsell E.F. (1992). High-performance liquid chromatography of oligosaccharide alditols and glycopeptides on a graphitized carbon column. Journal of Chromatography A.

[bb0050] De Leoz M.L.A., Gaerlan S.C., Strum J.S., Dimapasoc L.M., Mirmiran M., Tancredi D.J., Underwood M.A. (2012). Lacto-N-tetraose, fucosylation, and secretor status are highly variable in human milk oligosaccharides from women delivering preterm. Journal of Proteome Research.

[bb0055] De Leoz M.L.A., Kalanetra K.M., Bokulich N.A., Strum J.S., Underwood M.A., German J.B., Lebrilla C.B. (2015). Human milk glycomics and gut microbial genomics in infant feces show a correlation between human milk oligosaccharides and gut microbiota: A proof-of concept study. Journal of Proteome Research.

[bb0060] Dell A., Reason A.J., Khoo K.H., Panico M., McDowell R.A., Morris H.R. (1994). Mass spectrometry of carbohydrate-containing biopolymers. Methods in Enzymology.

[bb0065] Elwakiel M., Hageman J.A., Wang W., Szeto I.M., van Goudoever J.B., Hettinga K.A., Schols H.A. (2018). Human milk oligosaccharides in colostrum and mature milk of Chinese mothers: Lewis positive secretor subgroups. Journal of Agricultural and Food Chemistry.

[bb0070] Gidrewicz D.A., Fenton T.R. (2014). A systematic review and meta-analysis of the nutrient content of preterm and term breast milk. BMC Pediatrics.

[bb0075] Hageman M.E.J.A., Wang W., Szeto I.M., van Goudoever J.B., Hettinga K.A., Schols H.A. (2018). Human milk oligosaccharides in colostrum and mature milk of Chinese mothers: Lewis positive secretor subgroups. Journal of Agricultural and Food Chemistry.

[bb0080] Huang C., Sun S., Yan J., Wang H., Zhou J., Gao H., Xie W., Li Y., Chai W. (2020). Identification of carbohydrate peripheral epitopes important for recognition by positive-ion MALDI multistage mass spectrometry. Carbohydrate Polymers.

[bb5000] Huang C., Hou M., Yan J., Wang H., Wang Y., Cao C., Wang Y., Gao H., Ma X., Zheng Y., Bu D., Chai W., Li Y., Sun S. (2023). GIPS-mix for accurate identification of isomeric components in glycan mixtures using intelligent group-opting strategy. Analytical Chemistry.

[bb0085] Jantscher-Krenn E., Zherebtsov M., Nissan C., Goth K., Guner Y.S., Naidu N., Bode L. (2012). The human milk oligosaccharide disialyllacto-N-tetraose prevents necrotizing enterocolitis in neonatal rats. Gut.

[bb0095] Koizumi K., Okada Y., Fukuda M. (1991). High-performance liquid chromatography of mono-and oligosaccharides on a graphitized carbon column. Carbohydrate Research.

[bb0100] Labbok M., Clark D., Goldman A. (2004). Breastfeeding: Maintaining an irreplaceable immunological resource. Nature Reviews Immunology.

[bb0105] Lang Y., Zhang Y., Wang C., Huang L., Liu X., Song N., Li G., Yu G. (2021). Comparison of different labeling techniques for the LC-MS profiling of human milk oligosaccharides. Frontiers in Chemistry.

[bb0110] Leung A., Sauve R. (2005). Breast is best for babies. Journal of the National Medical Association.

[bb0115] Li J., Bi Y., Zheng Y., Cao C., Yu L., Yang Z., Chai W., Yan J., Lai J., Liang X. (2022). Development of high-throughput UPLC-MS/MS using multiple reaction monitoring for quantitation of complex human milk oligosaccharides and application to large population survey of secretor status and Lewis blood group. Food Chemistry.

[bb0120] Mank M., Hauner H., Heck A.J.R., Stahl B. (2020). Targented LC-ESI-MS2-characterization of human milk oligosaccharide diversity at 6 to 16 weeks post-partum reveals clear staging effects and distinctive milk groups. Analytical and Bioanalytical Chemistry.

[bb0125] Marx C., Bridge R., Wolf A.K., Rich W., Kim J.H., Bode L. (2014). Human milk oligosaccharide composition differs between donor milk and mother’s own milk in the NICU. Journal of Human Lactation.

[bb0130] Morrow A.L., Meinzen-Derr J., Huang P., Schibler K.R., Cahill T., Keddache M., Jiang X. (2011). Fucosyltransferase 2 non-secretor and low secretor status predicts severe outcomes in premature infants. Journal of Pediatrics.

[bb0135] Musilova S., Rada V., Vlkova E., Bunesova V. (2014). Beneficial effects of human milk oligosaccharides on gut microbiota. Beneficial Microbes.

[bb0140] Newburg D.S., Grave G. (2014). Recent advances in human milk glycobiology. Pediatric Research.

[bb0145] Newburg D.S., Ruiz-palacios G.M., Morrow A.L. (2005). Human milk glycans protect infants against enteric pathogens. Annual Review of Nutrition.

[bb0150] Pausan M.-R., Kolovetsiou-Kreiner V., Richter G.L., Madl T., Giselbrecht E., Obermayer-Pietsch B., Moissl-Eichinger C. (2020). Human milk oligosaccharides modulate the risk for preterm birth in a microbiome-dependent and -independent manner. mSystems.

[bb0155] Pendu J.L. (2004). Histo-blood group antigen and human milk oligosaccharides: Genetic polymorphism and risk of infectious diseases. Advances in Experimental Medicine Biology.

[bb0160] Perme M.P., Manevski D. (2019). Confidence intervals for the Mann-Whitney test. Statistical Methods in Medical Research.

[bb0165] Powe C.E., Puopolo K.M., Newburg D.S., Lonnerdal B., Chen C., Allen M., Welt C.K. (2011). Effects of recombinant human prolactin on breast milk composition. Pediatrics.

[bb0170] Redmond J.W., Packer N.H. (1999). The use of solid-phase extraction with graphitised carbon for the fractionation and purification of sugars. Carbohydrate Research.

[bb0175] Robinson R.C., Colet E., Tian T., Poulsen N.A., Barile D. (2018). An improved method for the purification of milk oligosaccharides by graphitized carbon-solid phase extraction. International Dairy Journal.

[bb0180] Senizza B., Ganugi P., Trevisan M., Lucini L. (2023). Combining untargeted profiling of phenolics and sterols, supervised multivariate class modelling and artificial neutral networks for the origin and authenticity of extra-virgin olive oil: A case study on Taggiasca Ligure. Food Chemistry.

[bb0185] Sun S., Huang C., Wang Y., Liu Y., Zhang J., Zhou J., Gao F., Yang F., Chen R., Mulloy B., Chai W., Li Y., Bu D. (2018). Toward automated identification of glycan branching patterns using multistage mass spectrometry with intelligent precursor selection. Analytical Chemistry.

[bb6000] Sun D., Hu F., Gao H., Song Z., Wang P., Shi L., Wang K., Li Y., Huang C., Li Z. (2019). Distribution of abnormal IgG glcosylation patterns from rheumatoid arthritis and osteoarthritis patients by MALDI-TOF-MSn. Analyst.

[bb0190] Theodorsson-Norheim E. (1986). Kruskal Kruskal-Wallis test: BASIC computer program to perform nonparametric one-way analysis of variance and multiple comparisons on ranks of several independent samples. Computer Methods and Programs in Biomedicine.

[bb0195] Totten S.M., Wu L.D., Packer E.A., Davis J.C.C., Hua S., Stroble C., Lebrilla C.B. (2014). Rapid-throughput glycomics applied to human milk oligosaccharide profiling for large human studies. Analytical and Bioanalytical Chemistry.

[bb0200] Underwood M.A., Gaerlan S., De Leoz M.L., Dimapasoc L., Kalanetra K.M., Lemay D.G., Lebrilla C.B. (2015). Human milk oligosaccharides in premature infants: Absorption, excretion, and influence on the intestinal microbiota. Pediatric Research.

[bb0205] van Leeuwen S.S., Schoemaker R.J.W., Gerwig G.J., van Leusen-van Kan E.J.M., Dijkhuizen L., Kamerling J.P. (2014). Rapid milk group classification by 1H NMR analysis of Le and H epitopes in human milk oligosaccharide donor samples. Glycobiology.

[bb0210] Wang B. (2012). Molecular mechanisms underlying sialic acid as essential nutrient for brain development and cognition. Advances in Nutrition.

