# Supplementary material for: Human milk oligosaccharides in milk of mothers with term and preterm delivery at different lactation stage
Source: Carbohydr Polym. 2023 Dec 1;321:121263. doi: 10.1016/j.carbpol.2023.121263 (PMC10565836; doi:10.1016/j.carbpol.2023.121263)
Supplement: Supplementary file 1 — Supplementary material [file mmc1.pdf]

Human milk oligosaccharides in milk of mothers with term and preterm delivery at different lactation stage

Chuncui Huang[1,4], Yue Lu[2], Lin Kong[2], Zhendong Guo[1,3], Keli Zhao[1], Zheng Xiang[2], Xinyue Ma[1,3], Huanyu Gao[1,4], Yongfang Liu[2], Zhongmin Gao[2], Lijuan Xu[2], Wengang Chai[5], Yan Li[1,2,3,4]*, Yao Zhao[2,]*

[1] Key Laboratory of Epigenetic Regulation and Intervention, Chinese Academy of Sciences, Institute of Biophysics, Chinese Academy of Sciences, 15 Datun Road, Beijing 100101, China.

[2] National Clinical Research Center for Child Health and Disorders, China International Science and Technology Cooperation base of Child development and Critical Disorders, Ministry of Education Key Laboratory of Child Development and Disorders, Chongqing Key Laboratory of Child Infection and Immunity, Children's Hospital of Chongqing Medical University, Chongqing, 400015, China.

[3] University of Chinese Academy of Sciences, 19 Yuquan Road, Beijing 100049, China. [4] Western Institute of Health Data Science, 28 High Tech Avenue, Chongqing 401329, China. [5] Glycosciences Laboratory, Faculty of Medicine, Imperial College London, London W12 0NN, United Kingdom.

*Corresponding Author

Email: yanli@ibp.ac.cn (Tel: 86-10-64887212), Zhaoy@cqmu.edu.cn (Tel: 86-10-64887212)

Supplementary Information

Table of Contents:

Supplementary text

Table S1. Removal rates of proteins in human milk using different extraction columns.

Table S2. Detected HMOs extracted from human milk using graphitic carbon cartridges and graphitic carbon 96-well plates.

Table S3. Comparison of relative intensity of HMOs from human milk extracted using graphitic carbon cartridges and graphitic carbon 96-well plates.

Table S4. The reproducibility of relative intensity of representative HMOs in the MALDI-MS spectra.

Table S5. HMOs with statistical differences between secretors and nonsecretors for women delivering preterm and term infants.

Figure S1. Removal rates of proteins in human milk using different extraction columns.

Figure S2. MALDI-TOF MS profiles of HMOs extracted using graphitized carbon 96-well plates and HMOs extracted without using graphitized carbon 96-well plates.

Figure S3. Relative intensity of HMOs in the MALDI-TOF MS spectra.

Figure S4. Significantly different HMOs detected in secretors for women with preterm delivery and term delivery.

Figure S5. Significantly different HMOs between different phenotypes for women with term delivery (a) and women with preterm delivery (b).

Figure S6. Discriminant analysis on different lactation stages.

Figure S7. Detailed dynamic changes of DSLNT during lactation for women with different gestation times.

Figure S8. Comparison of changes in sialylated HMOs, fucosylated HMOs and non-fucosylated neutral HMOs during lactation between secretors and nonsecretors.

Figure S9. Discriminant analysis on different secretor status (a), and distinctive HMOs for secretors and nonsecretors in different lactation stages (b).

Figure S10. Analysis on the value of variable importance for the projection (VIP) for specific HMOs for the differentiation of secretors and nonsecretors.

Supplementary text

OPLA-DA assay showed that secretors and nonsecretors were clearly differentiated through analysis of HMOs (Figure S9a), and seven HMOs were characterized for high VIP values to distinguish secretors and nonsecretors (Figure S10). These results demonstrated that HMOs were different between secretors and nonsecretors, and further study of different HMOs during lactation between secretors and nonsecretors were performed.

As shown in Figure S9b, in colostrum, HMOs attached with multiple fucose residues including H4N2F3, H4N2F4, H4N2F4S1 and H5N3F4 were significantly different between secretors and nonsecretors. In addition, H4N2F3, H5N3F1 H4N2F4 and H5N3F4 differed significantly in transitional milk from secretors and nonsecretors. We also found that H4N2F3, H4N2F4, H4N2F2S1 and H5N3F4 in mature milk were significantly different for women with different secretor status. Notably, three multiply fucosylated HMOs including H4N2F3, H4N2F4 and H5N3F4 were different in all lactation stages between secretors and nonsecretors, and the sialylated HMO, H4N2F4S1, was more abundant in both colostrum and mature milk from secretors than that in colostrum and mature milk from nonsecretors. As a result, dietary Sia which is important for immune response and brain development is suggested to be supplemented for breast-feeding infants from nonsecretors. Together, these observed differences in fucosylated and sialylated HMOs were in agreement with the result that the relative abundance of fucosylated and sialylated HMOs was different for secretors and nonsecreors (Figure S8).

Table S1. Removal rates of proteins in human milk using different extraction columns.

**Extraction columns**	**Removal rate (%, n=3)**		
	**Defatted (%)**	**After precipitation**	**After SPE extraction**
Graphitized carbon column	0	74.35±3.64	96.44±0.18
Graphitized carbon 96-well plate	0	73.92±1.12	93.20±0.33
C18- Graphitized carbon column	0	75.52±4.35	96.59±0.71
C18- Graphitized carbon 96-well plate	0	69.86±0.39	93.16±0.34

Table S2. Detected HMOs from human milk extracted using graphitic carbon cartridges and graphitic carbon 96-well plates.

**Permethylated [MNa$^+$]**	**Composition**	**Detected**	
		**Graphitized carbon column**	**Graphitized carbon 96-well plate**
825.41	H2F2	+	+
896.45[c]	H2N1F1	+	+
926.46	H3N1	+	+
1100.55	H3N1F1	+	+
1130.56	H4N1	+	+
1274.64	H3N1F2	+	+
1287.63	H3N1S1	+	+
1304.65	H4N1F1	+	+
1345.67	H3N2F1	+	+
1375.68	H4N2	+	+
1478.73	H4N1F2	+	+
1549.77	H4N2F1	+	+
1579.78	H5N2	+	+
1648.80	H3N1S2	+	+
1723.86	H4N2F2	+	+
1736.86	H4N2S1	+	+
1753.87	H5N2F1	+	+
1824.91	H5N3	+	+
1897.95	H4N2F3	+	+
1910.95	H4N2F1S1	+	+
1927.96	H5N2F2	+	+
1999.00	H5N3F1	+	+
2072.04	H4N2F4	+	+
2085.03	H4N2F2S1	+	+
**2098.03**	**H4N2S2**	**-**	**+**
2102.05	H5N2F3	+	+
2173.09	H5N3F2	+	+
2203.10	H6N3F1	+	+
**2272.12**	**H4N2F1S2**	**-**	**+**
2274.14	H6N4	+	+
2347.18	H5N3F3	+	+
2360.17	H5N3F1S1	+	+
2377.19	H6N3F2	+	+

2448.22	H6N4F1	+	+
2521.27	H5N3F4	+	+
2534.26	H5N3F2S1	+	+
2622.31	H6N4F2	+	+
2796.40	H6N4F3	+	+
**2809.40**	**H6N4F1S1**	**-**	**+**
2897.45	H7N5F1	+	+
2970.49	H6N4F4	+	+
3071.54	H7N5F2	+	+

Table S3. Comparison of relative intensity of HMOs from human milk extracted using graphitic carbon columns and graphitic carbon 96-well plates.

**Permethylated [MNa⁺]**	**Composition**	**Relative intensity (%)**		***p* Value**
		**Graphitized carbon column**	**Graphitized carbon 96-well plate**	
1274	H3N1F2	3.55±1.90	2.51±0.85	0.435
**1287**	**H3N1S1**	**0.39±0.28**	**1.58±0.50**	**0.023**
1375	H4N2	2.56±1.68	4.51±0.85	0.147
1549	H4N2F1	24.10±4.43	25.87±4.29	0.644
1648	H3N1S2	0.14±0.12	1.80±1.18	0.071
1723	H4N2F2	31.07±1.92	25.70±4.18	0.113
**1736**	**H4N2S1**	**0.76±0.40**	**2.78±0.43**	**0.004**
1824	H5N3	0.54±0.25	0.77±0.24	0.311
**1897**	**H4N2F3**	**8.71±1.21**	**5.22±0.23**	**0.008**
**1910**	**H4N2F1S1**	**2.34±1.20**	**5.92±0.37**	**0.008**
1999	H5N3F1	5.14±1.50	4.91±0.76	0.820
2173	H5N3F2	8.86±1.67	7.54±1.13	0.320
2347	H5N3F3	5.32±1.65	3.56±0.74	0.167
2448	H6N4F1	2.00±0.61	2.53±1.01	0.477
2622	H6N4F2	3.03±0.56	3.13±0.87	0.878

Table S4. The reproducibility of relative intensity of representative HMOs in the MALDI-MS spectra.

**Permethylated [MNa$^+$]**	**Composition**	**Relative intensity±SD (%, n=10)**	**Relative standard deviation of relative intensity (%RSD, n=10)**
1274	H3N1F2	7.13±0.78	10.89
1287	H3N1S1	0.12±0.01	5.35
1375	H4N2	0.72±0.09	12.23
1549	H4N2F1	29.53±10.26	10.26
1723	H4N2F2	39.12±3.10	10.77
1736	H4N2S1	0.16±0.18	14.13
1897	H4N2F3	1.48±0.16	10.76
1999	H5N3F1	3.46±0.48	13.76
2173	H5N3F2	10.36±1.41	13.59
2347	H5N3F3	4.66±0.64	13.81

Table S5. HMOs with statistical differences between secretors and nonsecretors for women delivering preterm and term infants.

**Permethylated [MNa$^+$]**	**Composition**	**Delivery**	**Relative abundance (%)**		***p* value**	**VIP value**
			**Se+**	**Se-**		
1274	H3N1F2	Preterm	2.13	1.00	0.001	1.13
1549	H4N2F1	Preterm	15.10	21.26	0.008	1.10
1897	H4N2F3	Preterm	5.52	0.86	<0.001	1.67
		Term	4.53	1.26	0.001	1.73
1999	H5N3F1	Preterm	7.69	11.09	<0.001	1.56
2072	H4N2F4	Preterm	0.34	0.05	<0.001	1.42
		Term	0.22	0.03	0.003	1.83
2085	H4N2F2S1	Preterm	1.10	0.17	<0.001	1.44
2173	H5N3F2	Preterm	10.80	16.85	0.001	1.46
2521	H5N3F4	Preterm	0.84	0.06	<0.001	1.71
		Term	0.23	0.03	0.016	1.57

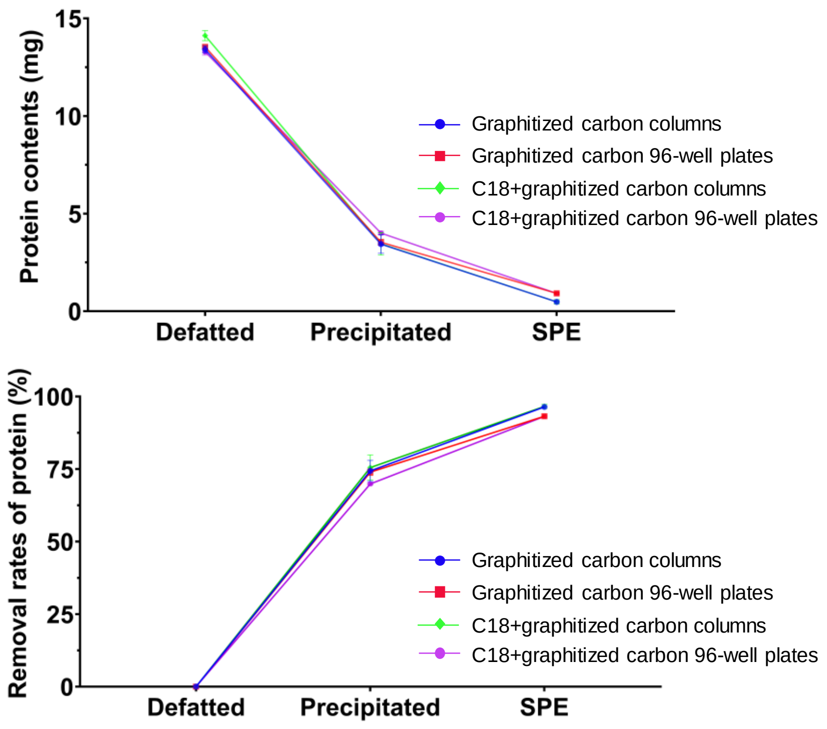

Figure S1. Removal rates of proteins in human milk using different extraction columns. The removal rates were evaluated in defatted milk, skim milk after precipitation using ethanol, and milk after SPE extraction.

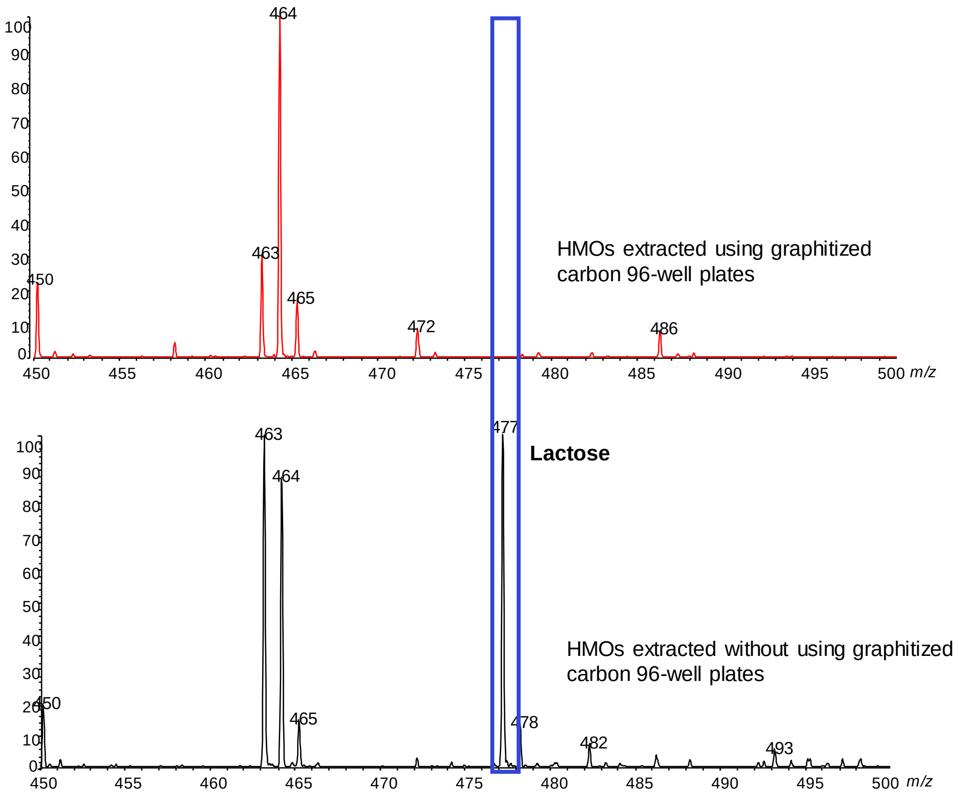

Figure S2. MALDI-TOF MS profiles of HMOs extracted using graphitized carbon 96-well plates and HMOs extracted without using graphitized carbon 96-well plates.

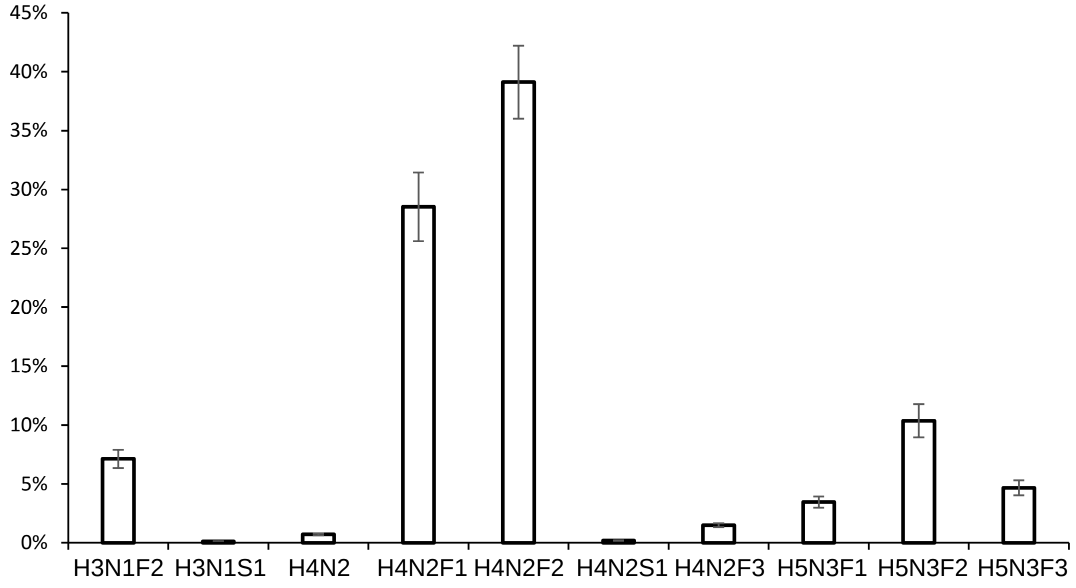

Figure S3. Relative intensity of HMOs in the MALDI-TOF MS spectra. Error bars represent the standard deviation (SD) of all the analysis (n=10).

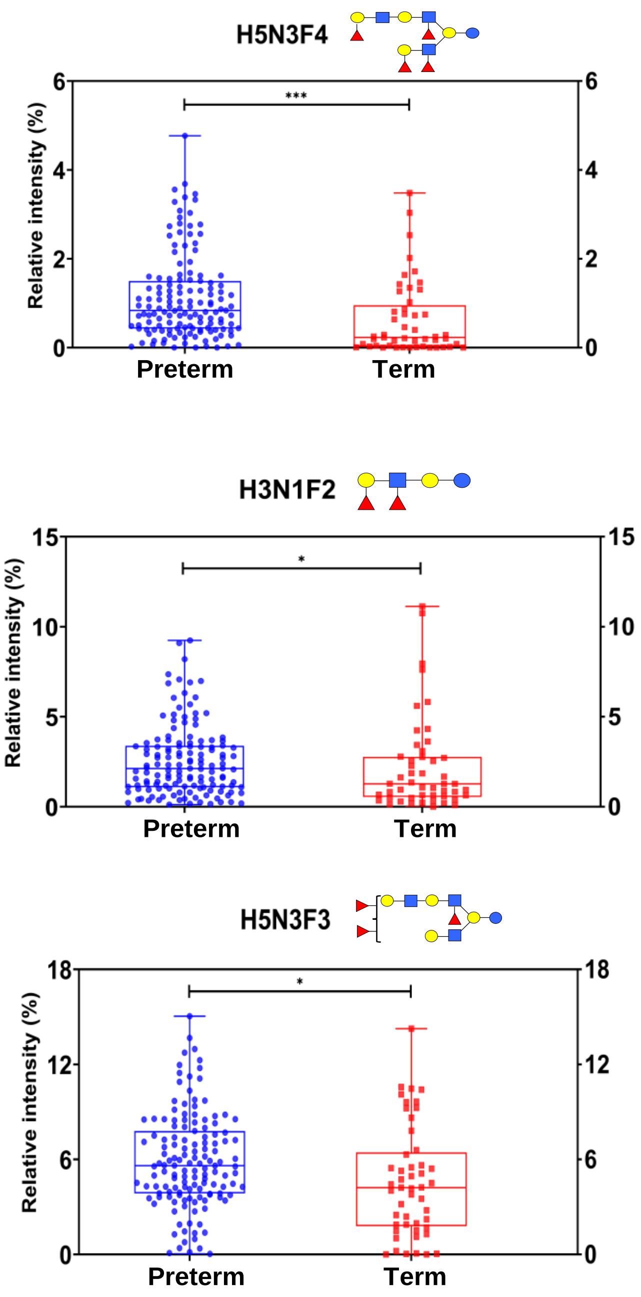

Figure S4. Significantly different HMOs detected in secretors for women with preterm delivery and term delivery.

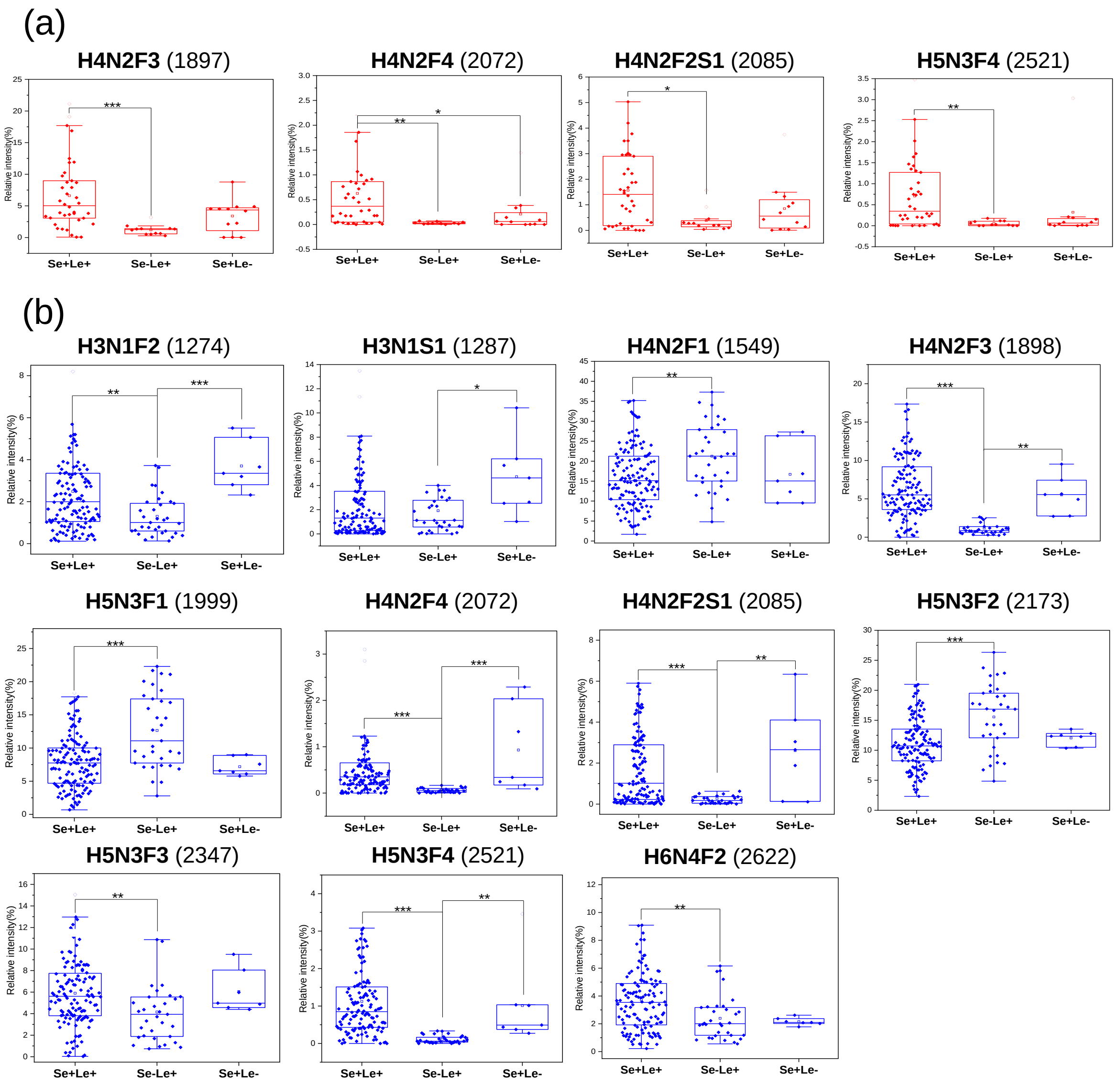

Figure S5. Significantly different HMOs between different phenotypes for women with term delivery (a) and women with preterm delivery (b).

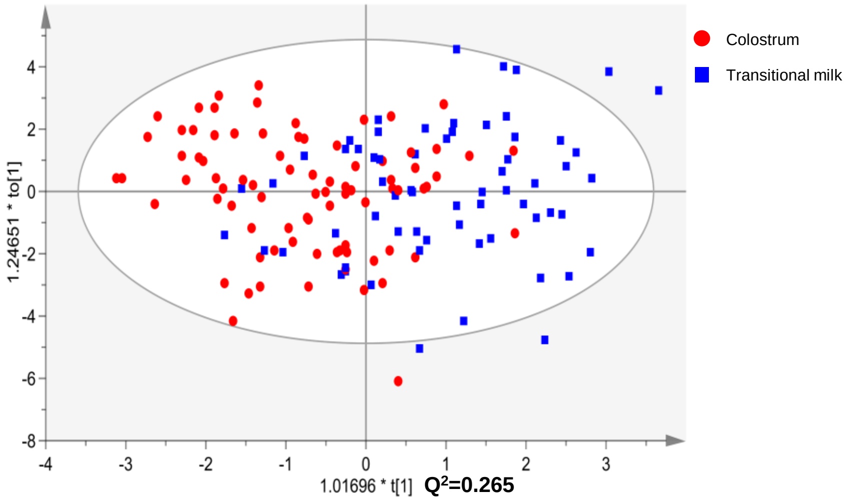

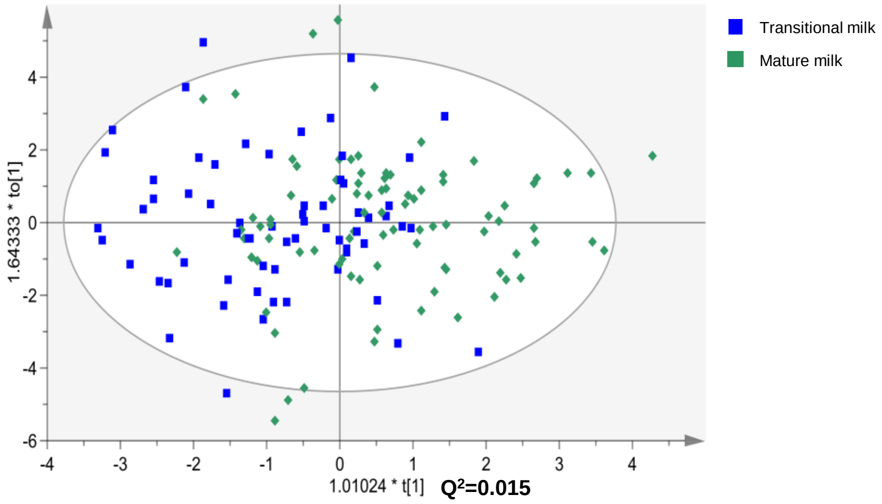

Figure S6. Discriminant analysis on different lactation stages. The OPLS-DA analysis was based on the 20 most abundant oligosaccharides in the milk.

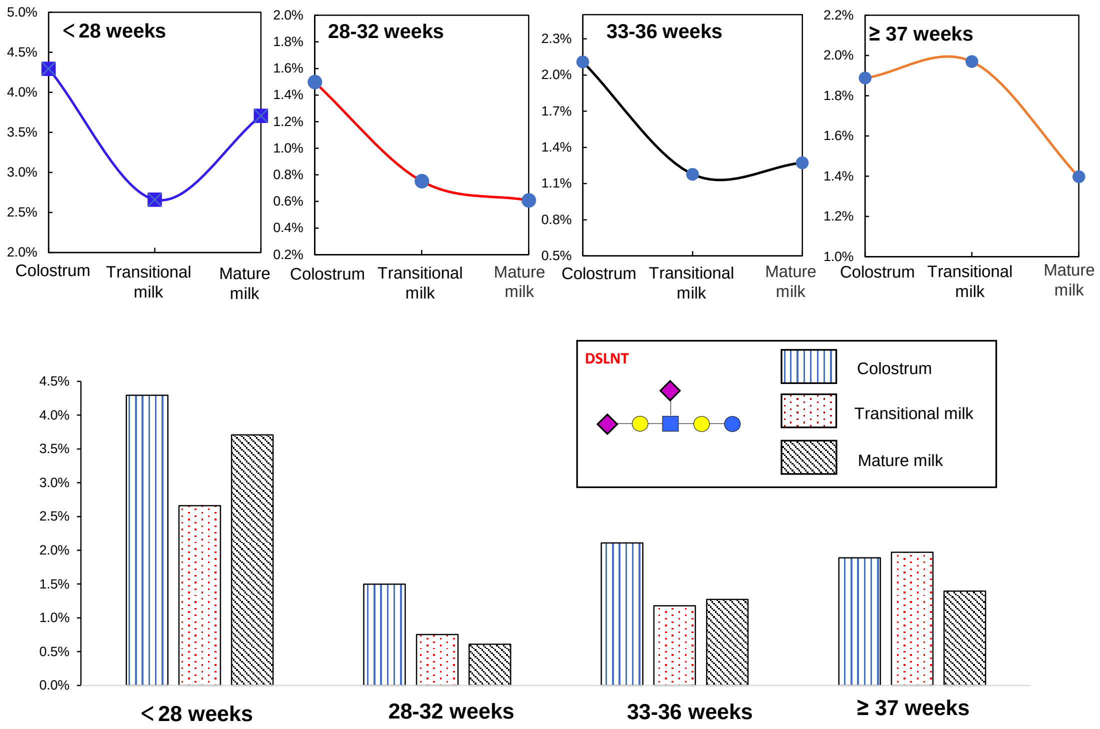

Figure S7. Detailed dynamic changes of DSLNT during lactation for women with different gestation times.

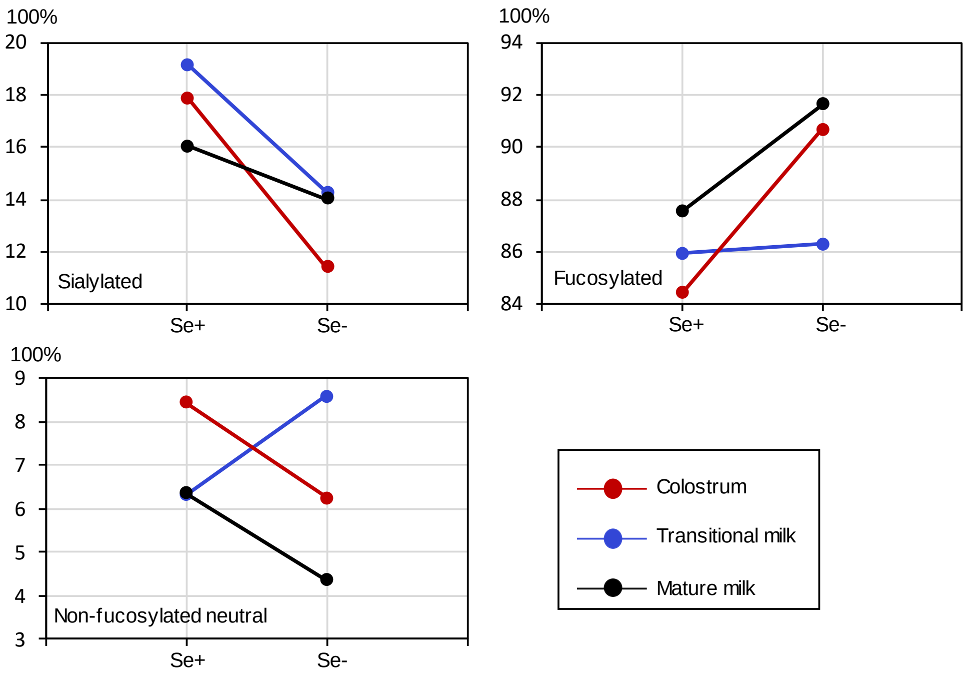

Figure S8. Comparison of changes in sialylated HMOs, fucosylated HMOs and non-fucosylated neutral HMOs during lactation between secretors and nonsecretors.

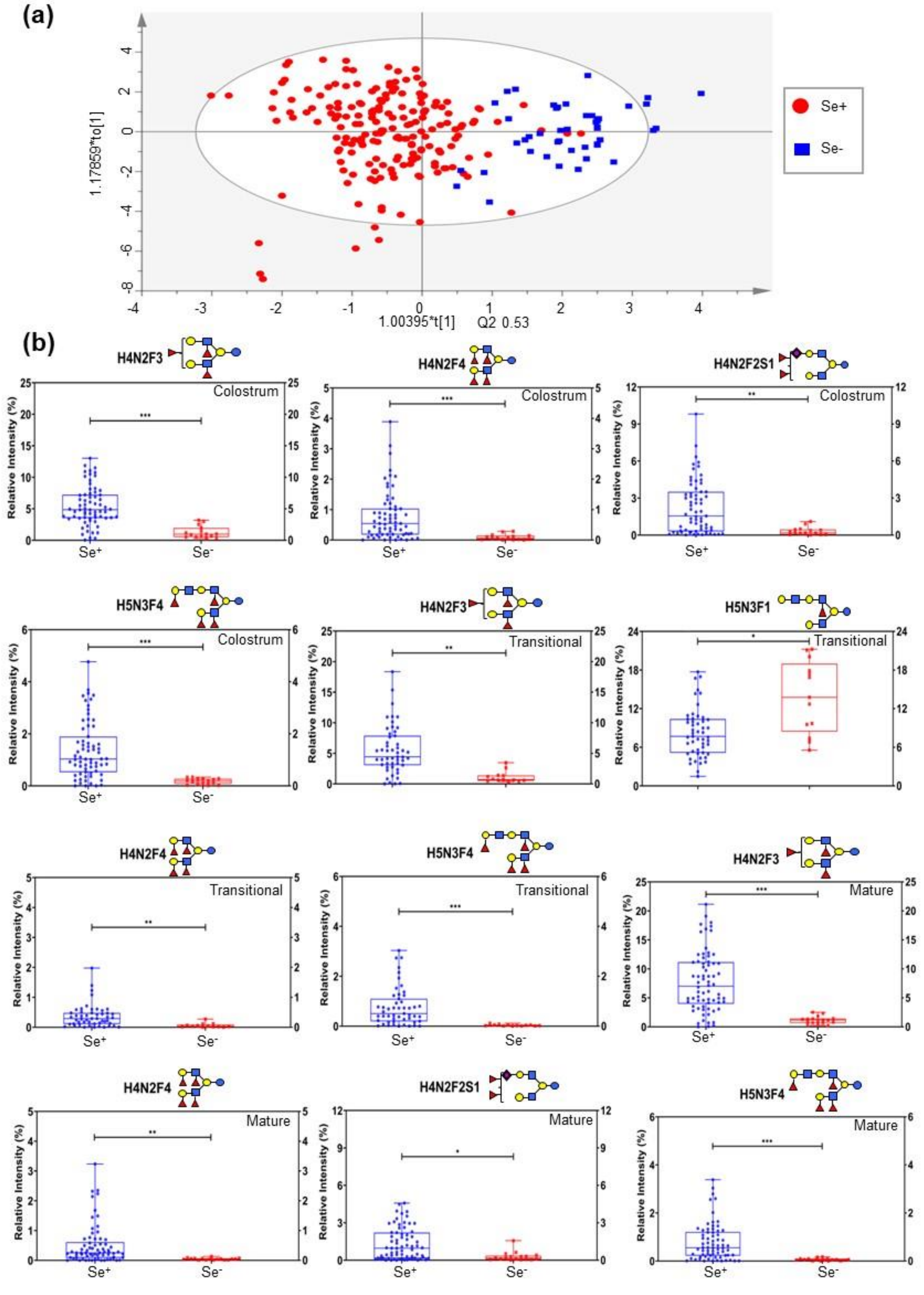

Figure S9. Discriminant analysis on different secretor status (a), and distinctive HMOs for secretors and nonsecretors in different lactation stages (b).

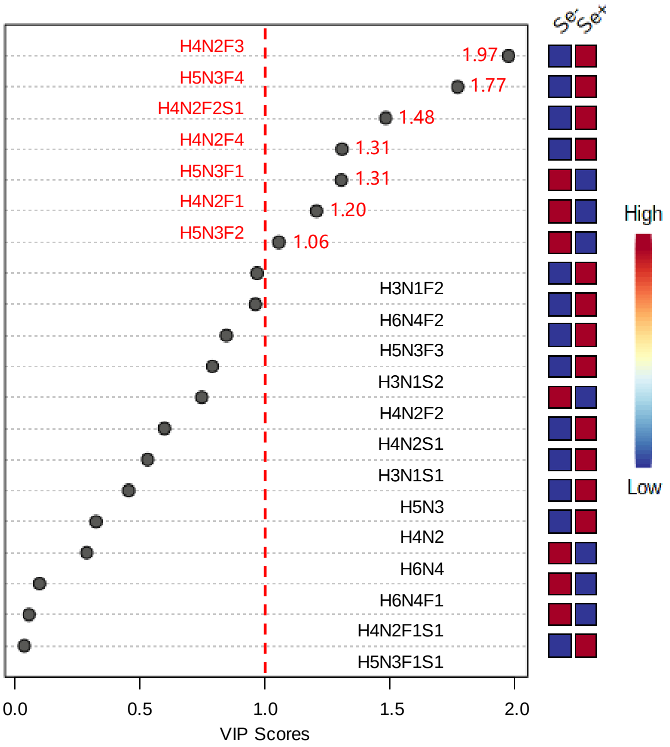

Figure S10. Analysis on the value of variable importance for the projection (VIP) for specific HMOs for the differentiation of secretors and nonsecretors.
